# Wide Distribution of Phage That Infect Freshwater SAR11 Bacteria

**DOI:** 10.1128/mSystems.00410-19

**Published:** 2019-10-22

**Authors:** Lin-Xing Chen, Yanlin Zhao, Katherine D. McMahon, Jiro F. Mori, Gerdhard L. Jessen, Tara Colenbrander Nelson, Lesley A. Warren, Jillian F. Banfield

**Affiliations:** a Department of Earth and Planetary Sciences, University of California, Berkeley, California, USA; b Fujian Provincial Key Laboratory of Agroecological Processing and Safety Monitoring, College of Life Sciences, Fujian Agriculture and Forestry University, Fuzhou, Fujian, China; c Department of Civil and Environmental Engineering, University of Wisconsin, Madison, Wisconsin, USA; d Department of Civil and Mineral Engineering, University of Toronto, Toronto, Canada; e School of Geography and Earth Science, McMaster University, Hamilton, Canada; f Earth Sciences Division, Lawrence Berkeley National Laboratory, Berkeley, California, USA; g Department of Environmental Science, Policy, and Management, University of California, Berkeley, California, USA; h Chan Zuckerberg Biohub, San Francisco, California, USA; i Innovative Genomics Institute at UC Berkeley, Berkeley, California, USA; j The University of Melbourne, Melbourne, Australia; k Department of Bacteriology, University of Wisconsin, Madison, Wisconsin, USA; University of Hawaii at Manoa

**Keywords:** SAR11, LD12, *Fonsibacter*, freshwater phage, Pelagiphage, genome-resolved metagenomics

## Abstract

*Fonsibacter* (LD12 subclade) is among the most abundant bacterioplankton in freshwater ecosystems. These bacteria belong to the order *Pelagibacterales* (SAR11) and are related to *Pelagibacter* (marine SAR11), which dominates many marine habitats. Although a few *Pelagibacter* phage (Pelagiphage) have been described, no phage that infect *Fonsibacter* have been reported. In this study, we describe two groups of *Podoviridae* phage that infect *Fonsibacter*. A complete *Fonsibacter* genome containing a prophage was reconstructed from metagenomic data. A circularized and complete genome related to the prophage, referred to as uv-Fonsiphage-EPL (lysogenic strategy), shows high similarity to marine Pelagiphage HTVC025P. Additionally, we reconstructed three complete genomes and one draft genome of phage related to marine Pelagiphage HTVC010P and predicted a lytic strategy. The similarity in codon usage and cooccurrence patterns of HTVC010P-related phage and *Fonsibacter* suggested that these phage infect *Fonsibacter*. Similar phage were detected in Lake Mendota, Wisconsin, where *Fonsibacter* is also present. A search of related phage revealed the worldwide distribution of some genotypes in freshwater ecosystems, suggesting their substantial role in shaping indigenous microbial assemblages and influence on biogeochemical cycling. However, the uv-Fonsiphage-EPL and one group of HTVC010P-related phage have a more limited distribution in freshwater ecosystems. Overall, the findings provide insights into the genomic features of phage that infect *Fonsibacter* and expand understanding of the ecology and evolution of these important bacteria.

**IMPORTANCE** *Fonsibacter* represents a significant microbial group of freshwater ecosystems. Although the genomic and metabolic features of these bacteria have been well studied, no phage infecting them has been reported. In this study, we reconstructed complete genomes of *Fonsibacter* and infecting phage and revealed their close relatedness to the phage infecting marine SAR11 members. Also, we illustrated that phage that infect *Fonsibacter* are widely distributed in freshwater habitats. In summary, the results contribute new insights into the ecology and evolution of *Fonsibacter* and phage.

## Abstract (precis)

*Fonsibacter* represents a significant microbial group of freshwater ecosystems. Although the genomic and metabolic features of these bacteria have been well studied, no phage infecting them has been reported. In this study, we reconstructed complete genomes of *Fonsibacter* and infecting phage and revealed their close relatedness to the phage infecting marine SAR11 members. Also, we illustrated that phage that infect *Fonsibacter* are widely distributed in freshwater habitats. In summary, the results contribute new insights into the ecology and evolution of *Fonsibacter* and phage.

## INTRODUCTION

Heterotrophic SAR11 bacteria (*Alphaproteobacteria*; *Pelagibacterales*) are often very abundant in marine and freshwater ecosystems (1–4). *Fonsibacter*, the freshwater subclade of SAR11, also known as LD12 (or III-b), is especially abundant in the euphotic layers of lakes during summer (1) and plays important roles in the assimilation of low-molecular-weight carboxylic acids (1, 5). The first *Fonsibacter* genomes were reconstructed via single-cell genomics, and subsequent analyses indicated their low recombination rates in nature (3). Comparative genomic analyses showed many proteins shared between *Fonsibacter* and *Pelagibacter* (marine SAR11), but metabolic divergence was also detected (2). *Fonsibacter* typically uses the Embden-Meyerhof-Parnas (EMP) rather than the Entner–Doudoroff glycolysis pathway and produces rather than takes up osmolytes (5, 6). These studies proposed that *Fonsibacter* evolved from a streamlined ancestor of marine *Pelagibacter* (3, 5). It was initially proposed that the transition between marine and freshwater ecosystems happened only once (7), but this conclusion was challenged recently. For example, a metagenomics-assembled genome from the freshwater Lake Baikal was phylogenetically assigned to *Pelagibacter* (4), and phylogenetic analyses of 16S rRNA genes suggested the existence of several marine SAR11 subtypes in freshwater lakes (8). The first cultivated representative of *Fonsibacter* isolated from the southern Louisiana coast (9), reported very recently, has isocitrate lyase for a complete glyoxylate bypass of the tricarboxylic acid (TCA) cycle along with malate synthase, distinguishing it from other *Fonsibacter* bacteria. The authors suggest temperature-based ecotype diversification within this genus.

SAR11 rarely uses CRISPR-Cas or restriction-modification systems for phage defense (3, 10, 11). However, these bacteria harbor the hypervariable region 2 located between their 16S/23S rRNA and 5S rRNA genes, which contains genes encoding various transferases, isomerases, O antigen, and pilins. SAR11 may use these proteins to defend against phage by cell surface modification (2, 3, 9). To date, 15 *Pelagibacter* phage (Pelagiphages) have been isolated from marine environments (10, 12), and Pelagiphage HTVC010P is suggested to be among the most abundant phage in the ocean (10). In contrast, no phage that infect *Fonsibacter* have been reported. In general, phage that infect major heterotrophic groups in freshwater ecosystems are largely unknown, with only a few cases reported recently, including phage of the LD28 clade (“*Candidatus* Methylopumilus planktonicus”) (13) and the *Actinobacteria* acl clade (14). Phage that infect freshwater heterotrophic bacterial groups could shape the freshwater microbial assemblages and redistribute bacterially derived compounds via the lysis of host cells. Thus, phage of heterotrophic freshwater bacteria may significantly influence biogeochemical cycles, especially those of of carbon.

Here, we performed genome-resolved metagenomic analyses on microbial communities from freshwater ecosystems to reconstruct genomes of *Fonsibacter* bacteria and their phage. Comparative analyses of *Fonsibacter*- and *Pelagibacter*-infecting phage show genetic conservation and divergence. The distribution of some related phage in freshwater ecosystems suggests the broad ecological significance of *Fonsibacter* phage. Overall, the findings shed light on the ecology of *Fonsibacter* and reveal aspects of phage and host evolutionary history.

## RESULTS

### Metagenome-assembled genome of *Fonsibacter*.

**FIG 1:**
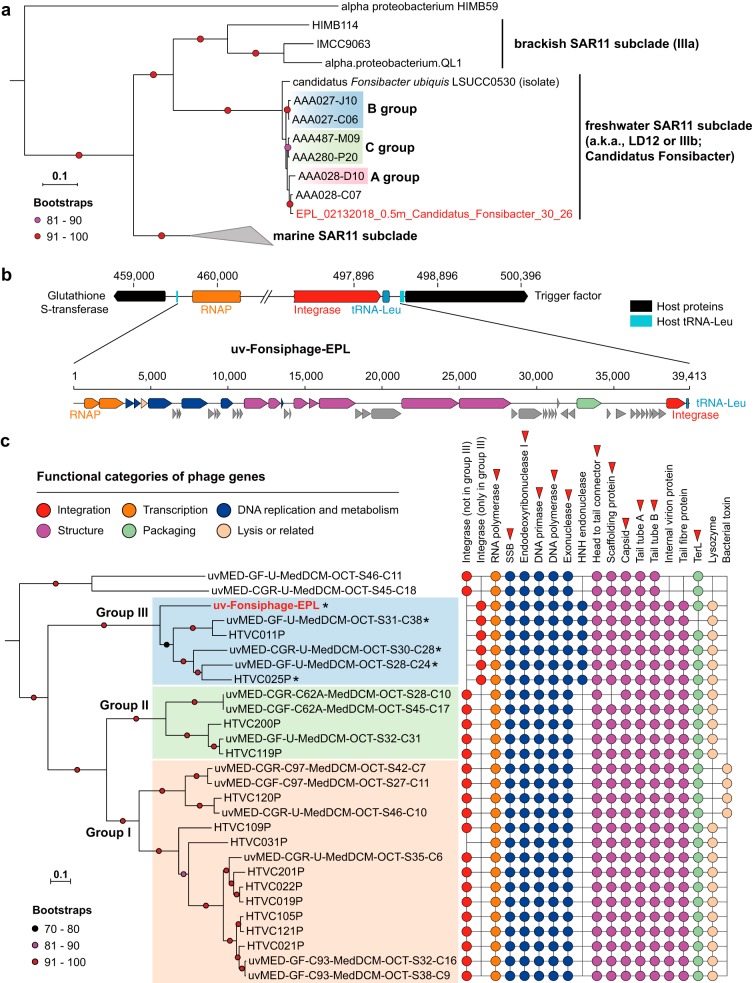
The complete *Fonsibacter* genome and its prophage. (a) Phylogenetic analyses of the complete *Fonsibacter* genome based on 16 ribosomal proteins (Materials and Methods). The three *Fonsibacter* groups defined previously are shown. The tree was rooted using the HIMB59 sequence. (b) The prophage of the complete *Fonsibacter* genome. The insertion site of the phage genome into the host tRNA-Leu is shown. Refer to panel c for the colors of different functional categories; hypothetical proteins are indicated in gray. (c) Phylogenetic analyses of uv-Fonsiphage-EPL and related phage based on 12 core proteins (red triangles) (Materials and Methods). The three *HTVC019Pvirus* groups defined recently are shown. The presence of protein families with predicted function in the phage is shown on the right (see Table S3 at https://figshare.com/articles/Supplementary_data_for_Chen_et_al_2019/9911318). The phage with a fragmented RNA polymerase is indicated by an asterisk. SSB, single-stranded DNA-binding protein.

Freshwater samples were collected from an end pit lake (EPL) in Alberta, Canada (see Materials and Methods). Analysis of the EPL metagenomic data sets (see Table S1 at https://figshare.com/articles/Supplementary_data_for_Chen_et_al_2019/9911318) revealed one genome bin with 27 scaffolds, two of which had features indicative of a prophage. Subsequently, this bin was manually curated into a complete genome. The genome accuracy was verified based on paired read mapping throughout. It contains no repeats long enough to have confounded the assembly and displays GC skew and cumulative GC skew with the form expected for complete bacterial genomes that undergo bidirectional replication (see Fig. S1 at https://figshare.com/articles/Supplementary_data_for_Chen_et_al_2019/9911318). The genome is 1,136,868 bp in length, the smallest SAR11 genome yet reported, and has a GC content of 29.6% (Table 1). Phylogenetic analyses based on a set of 16 ribosomal proteins indicated that the genomically defined bacterium belongs to the candidate genus *Fonsibacter* (Fig. 1a). The 16S rRNA gene sequence of this *Fonsibacter* genome is identical to that of AAA028-C07, recovered from Lake Mendota, Wisconsin (3), and shares 99.8% identity with that of the *Fonsibacter* isolate “*Candidatus* Fonsibacter ubiquis” LSUCC0530 (9). The new *Fonsibacter* genome shares 96% and 86% genome-wide average nucleotide identity (ANI) with the 0.85-Mbp AAA028-C07 draft and 1.16-Mbp complete LSUCC0530 genomes, respectively. We refer to the newly described complete genome as “EPL_02132018_0.5m_Candidatus_Fonsibacter_30_26” (here “*Fonsibacter*_30_26”).

**TABLE 1:** General features of the *Fonsibacter* and infecting phage genomes reconstructed in this study^*a*^

Genome (short name in the main text if provided)	Life strategy	Relative	Length (bp)	GC content (%)	Completeness (%)		No. of:		
					51 SCGs	CheckM	rRNAs	tRNAs	Proteins
EPL_02132018_0.5m_Candidatus_Fonsibacter_30_26^*b*^ (Fonsibacter_30_26)		AAA028-C07	1,136,868	29.6	50	100	3 (5S, 16S, 23S)	31	1,229
uv-Fonsiphage-EPL^*b*^	Lysogenic	Pelagiphage HTVC025P	39,413	32.1				1	52
EPL_06132017_6.25m_HTVC010P-related_33_76^*b*^ (HTVC010P-related_33_76)	Lytic	Pelagiphage HTVC010P	35,816	32.5					61
EPL_08022017_1.5m_HTVC010P-related_32_16^*b*^ (HTVC010P-related_32_16)	Lytic	Pelagiphage HTVC010P	36,457	31.9					60
I-EPL_09192017_0.5m_HTVC010P-related_33_10^*b*^ (HTVC010P-related_33_10)	Lytic	Pelagiphage HTVC010P	36,507	32.5					62
BMMRE_07242016_10_scaffold_124^*c*^	Lytic	Pelagiphage HTVC010P	27,140	31.6					33

a The draft genome of an HTVC010P-related phage from BMMRE (Materials and Methods) is also included, which could not be closed due to low sequencing coverage. The predicted life strategy and the closest reference for each phage are shown. Please note that the *Fonsibacter* genome lacks one of the 51 SCGs used for completeness evaluation; see the text and https://figshare.com/articles/Supplementary_data_for_Chen_et_al_2019/9911318 for details.

b Complete genome.

c Draft genome.

The *Fonsibacter*_30_26 genome includes 1,229 protein-encoding genes, 3 rRNA genes (one copy each of the 5S, 16S, and 23S rRNA genes), and 31 tRNAs (Table 1). *Fonsibacter*_30_26 does not include the gene for the 50S ribosomal protein L30, a feature that we predict is shared by all reported SAR11 genomes (see Fig. S2 at https://figshare.com/articles/Supplementary_data_for_Chen_et_al_2019/9911318). *Fonsibacter*_30_26 has the full EMP glycolysis pathway, a complete gluconeogenesis pathway, and a full TCA cycle and also a complete oxidative phosphorylation pathway and the nonoxidative pentose phosphate pathway, as reported for other *Fonsibacter* genomes (2, 3, 9). No carbon fixation gene or pathway was identified in the genome, indicating a heterotrophic lifestyle of this *Fonsibacter* species. Interestingly, within a region previously described to be hypervariable in SAR11 (53,690 bp in length in Fonsibacter_30_26, 54 protein-encoding genes [see Fig. S3 and S4 at https://figshare.com/articles/Supplementary_data_for_Chen_et_al_2019/9911318]), we detected four genes encoding transketolase, one of the three enzymes in the nonoxidative pentose phosphate pathway. However, these transketolases contained only one or two of the three domains found in a full-length transketolase sequence; therefore, their function in the pentose phosphate pathway remains uncertain. We identified 18 genes in the hypervariable region that encode glycosyltransferase, methyltransferase, and epimerase, which are common in SAR11 and may be involved in phage defense (2, 3, 9).

### The first genome of phage infecting *Fonsibacter*.

We mapped metagenomic reads to the putative *Fonsibacter*_30_26 prophage region and recovered reads that could be reconstructed into a complete phage genome (https://figshare.com/articles/Supplementary_data_for_Chen_et_al_2019/9911318). The prophage genome is inserted between *attL* (left end of prophage) and *attR* (right end of prophage), which share an 11-bp identical “core sequence.” Specifically, ∼5% of reads circularized the phage genome, indicating the presence of some free phage particles. In addition, some bacterial cells lack the prophage, so the prophage start and end could be clearly defined. We refer to the reconstructed sequence as uv-Fonsiphage-EPL. To our knowledge, this is the first genome of phage infecting *Fonsibacter*.

The genome of uv-Fonsiphage-EPL has a length of 39,413 bp and GC content of 32.1% and encodes 52 proteins (Table 1), including integrase, DNA metabolism, and replication gene products; phage structural gene products; the lysis gene product; and large terminase (TerL) (Fig. 1b). A search of the TerL sequence against the NCBI database revealed that uv-Fonsiphage-EPL is most closely related to phage from marine habitats. This was confirmed using phylogenetic analyses based on 12 core phage proteins (Fig. 1c; see also Tables S2 and S3 at https://figshare.com/articles/Supplementary_data_for_Chen_et_al_2019/9911318). In detail, uv-Fonsiphage-EPL grouped with two Pelagiphage isolates from the Baltic Sea (HTVC025P) (12) and Oregon coast seawater (HTVC011P) (10), and three metagenomically retrieved phage from the Mediterranean (15), within the *HTVC019Pvirus* (*Caudovirales*, *Podoviridae*, *Autographivirinae*) group III defined recently (12). uv-Fonsiphage-EPL shares 75.8 to 78.5% TerL similarity with group III members and is most similar to HTVC025P. Genome-wide alignment revealed conserved genome synteny and high similarity between uv-Fonsiphage-EPL and two Pelagiphage isolates (see Fig. S5a at https://figshare.com/articles/Supplementary_data_for_Chen_et_al_2019/9911318).

The phage in the *HTVC019Pvirus* group III share several features (Fig. 1c). For example, they have an integrase with a limited degree of identity with those in group I and II members and an HNH endonuclease that is absent in the other two groups. Although the integrases shared low similarities within group III (31.6 to 36.9%), uv-Fonsiphage-EPL, HTVC011P, and HTVC025P all can integrate into the host tRNA-Leu (TAG) site, and the core sequence (https://figshare.com/articles/Supplementary_data_for_Chen_et_al_2019/9911318) in uv-Fonsiphage-EPL is only 1 bp different from that of HTVC025P (12). The phage contains its own tRNA-Leu, replacing the lost function of the host tRNA-Leu gene after phage integration (Fig. 1b). A bacterial trigger factor protein flanks the prophage in all three host genomes (Fig. 1b) (12). Also, divergence was detected among *HTVC019Pvirus* group III members: for example, uv-Fonsiphage-EPL lacked several hypothetical proteins found in most of the other phage (see Table S3 at https://figshare.com/articles/Supplementary_data_for_Chen_et_al_2019/9911318).

Interestingly, all of the RNA polymerase genes in all group III genomes, except HTVC011P, were fragmented into two parts (Fig. 1c). We did not identify any RNA polymerase reads mapped to the uv-Fonsiphage-EPL genome that were not split. This, in combination with detection of the split gene in the other genomes, suggests that gene interruption did not occur recently.

### Metagenome-assembled genomes of potential *Fonsibacter-*infecting phage.

**FIG 2:**
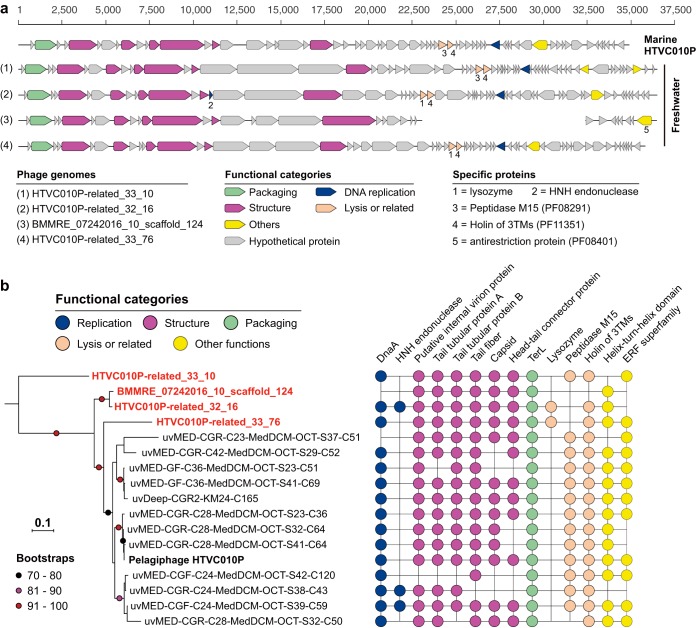
Pelagiphage HTVC010P-related phage genomes reconstructed in this study. (a) Gene content of the four phage genomes (three complete and one draft), compared to that of the marine Pelagiphage HTVC010P. Genes with predicted annotations are marked with different colors according to their function. The scaffold representing the BMMRE draft genome was split into two for better visual comparison of the genes. (b) Phylogenetic analyses of phage from this study (in red and boldface) and those from marine environments based on the TerL. For each genome, the presence of protein families with predicted function in the phage is shown on the right (see Table S4 at https://figshare.com/articles/Supplementary_data_for_Chen_et_al_2019/9911318).

Given the high similarity among uv-Fonsiphage-EPL and the *HTVC019Pvirus* Pelagiphage (see above), we expected to detect counterparts of other types of marine Pelagiphage in freshwater ecosystems (10). A subset of scaffolds from samples of EPL, I-EPL (the input source of EPL), and BMMRE, a base metal mine receiving environment in Manitoba, Canada (see Materials and Methods), encode TerL that shares 62 to 85% amino acids with Pelagiphage HTVC010P (*Podoviridae*). Manual curation generated three distinct complete genomes and one draft genome. These are referred to as HTVC010P-related phage (Table 1 and Fig. 2a) and showed genome-wide similarity with HTVC010P (see Fig. S5b at https://figshare.com/articles/Supplementary_data_for_Chen_et_al_2019/9911318). These phages may be in the stage of active infection and/or adsorption to the host’s cell surface, given that the cells in the communities were collected by filtering them onto filters with 0.2-μm pores (see Materials and Methods).

We identified phage-specific proteins in all the HTVC010P-related genomes, including DnaA, TerL, internal protein A, tail tubular proteins A and B, tail fiber, capsid, and head-tail connector protein (Fig. 2; see also Table S4 at https://figshare.com/articles/Supplementary_data_for_Chen_et_al_2019/9911318). No integrase was detected, suggesting that they are lytic phages. An HNH endonuclease was identified in HTVC010P-related_32_16 and two marine phage genomes but not in the BMMRE draft genome, though they are phylogenetically closely related (Fig. 2b). Interestingly, within all the HTVC010P-related genomes, only two of those reconstructed in this study harbored a lysozyme protein. Instead, those without lysozyme may use a peptidase M15 (PF08291) for the cell lysis function (https://figshare.com/articles/Supplementary_data_for_Chen_et_al_2019/9911318). No lysozyme or peptidase M15 was detected in the BMMRE draft genome, likely due to incompleteness. It is the only phage genome analyzed here that encodes a putative antirestriction protein (PF08401), possibly for protecting its DNA against host endonuclease activity. The HTVC010P-related phage genomes also shared genes encoding many hypothetical proteins (see Table S4 at https://figshare.com/articles/Supplementary_data_for_Chen_et_al_2019/9911318), suggesting their potentially important function.

### Evidence that HTVC010P-related phage infect *Fonsibacter*.

We speculated that the lytic HTVC010P-related phage could infect *Fonsibacter*, given their close relationship with Pelagiphage HTVC010P. Matches of spacers from CRISPR-Cas systems to the phage genome (16) and similar tRNA sequence (17) can be used to predict phage-host associations. Unfortunately, no CRISPR-Cas system was detected in *Fonsibacter*-related scaffolds in the EPL/I-EPL/BMMRE samples. Further, we did not detect any phylogenetically informative host-associated genes in the phage genomes that could indicate host range.

**FIG 3:**
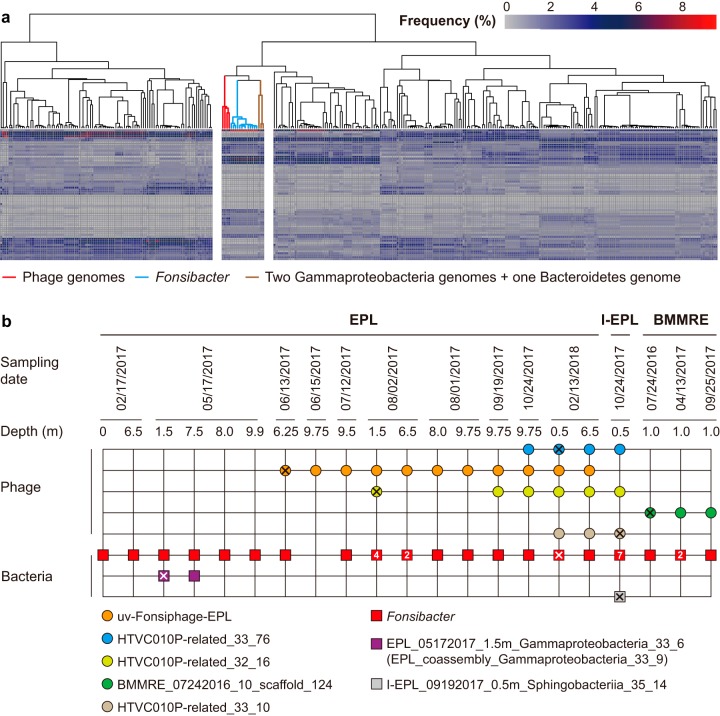
Evidence supporting the infection of *Fonsibacter* by HTVC010-related phage. (a) Clustering of genomes based on codon usage frequency. Each column represents a genome, and each line represents a codon type. The subclusters, including phage, *Fonsibacter*, and three other genomes, are separate. (b) The occurrence of phage (circles) and *Fonsibacter* and the three potential host bacteria (squares). All genotypes were detected once, unless a number is given inside the circle or square. The sample from which the phage or bacterial genomes were reconstructed is indicated by “X.”

Phage use host translational mechanisms during their lytic cycle (18), so they may adapt to host-preferred codons (19–21). Thus, codon usage bias is another approach to infer host-phage associations. We clustered all bacterial and archaeal and the four HTVC010P-related phage genomes from the same samples based on their codon usage frequency (see Table S5 at https://figshare.com/articles/Supplementary_data_for_Chen_et_al_2019/9911318). The results showed that the four phage clustered with all 13 *Fonsibacter* genomes (Fig. 3a), along with two *Gammaproteobacteria* and one *Bacteroidetes* genomes.

We evaluated the cooccurrence of *Fonsibacter*, the two *Gammaproteobacteria* and one *Bacteroidetes* species, and the phage in the EPL/I-EPL/BMMRE samples and found that all of the samples that contained HTVC010P-related phage had at least one *Fonsibacter* genotype (Fig. 3b; see also Fig. S6 at https://figshare.com/articles/Supplementary_data_for_Chen_et_al_2019/9911318). However, of the samples that contained HTVC010P-related phage, the *Bacteroidetes* species was detected in only the I-EPL sample. The two *Gammaproteobacteria* bacteria were detected in only two samples, neither of which contained the phage (Fig. 3b). In combination, the cooccurrence patterns strongly support the inference that the HTVC010P-related phage infect *Fonsibacter* bacteria. Some samples contained only one *Fonsibacter* type and multiple phage genotypes (e.g., EPL_02/13/2018_6.5m [Fig. 3b]), and some samples contained only one phage but multiple *Fonsibacter* types (e.g., BMMRE_04/13/2017_1.0m [Fig. 3b]). These findings indicate the “multiple versus multiple” host-phage relationship, in line with previous studies on *Pelagibacter* and its phage (10, 12, 15).

### *Fonsibacter* and its phage in Lake Mendota.

**FIG 4:**
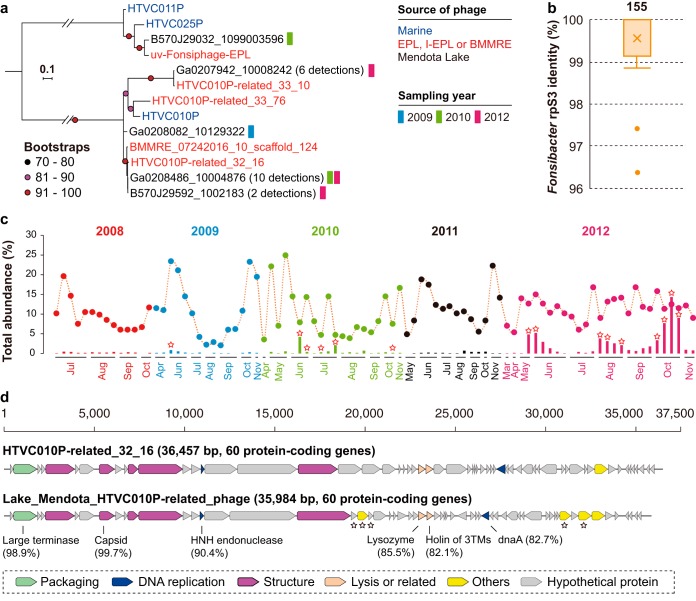
The occurrence of *Fonsibacter* and infecting phage in Lake Mendota. (a) Phylogenetic analyses of detected phage related to those from EPL/I-EPL/BMMRE in Lake Mendota based on the TerL protein. The number of genotypes is indicated in parentheses. Phage from marine habitats and those reconstructed in this study were included for reference. (b) The similarity of *Fonsibacter* rpS3 proteins between EPL/I-EPL/BMMRE and Lake Mendota. The total number of *Fonsibacter* rpS3 proteins is shown above the box plot. (c) The relative abundance of *Fonsibacter* (colored circles) and phage (colored bars) across the 5-year sampling period in Lake Mendota. A red-outlined star indicates the detection of assembled TerL in the corresponding metagenomic data set. (d) Comparative analyses of the phage genomes reconstructed from Lake Mendota (close to HTVC010P-related_32_16) and EPL (HTVC010P-related_32_16). The sequence similarities of some proteins between these two genomes are shown. A black-outlined star indicates genes not in the EPL phage genome.

To further investigate the potential distribution of *Fonsibacter* and its phage, we analyzed a time series metagenomic data set from Lake Mendota, where the first *Fonsibacter* genomes were reported (3). For this site, *Fonsibacter* strain dynamics were investigated over a 5-year period (22). A homolog search for TerL detected 19 HTVC010P-related TerL sequences in 14 of the 90 Lake Mendota samples (Fig. 4a; see also Table S6 at https://figshare.com/articles/Supplementary_data_for_Chen_et_al_2019/9911318). Eighteen of the TerL sequences shared ≥97% amino acid identity with sequences from HTVC010P-related_32_16 and HTVC010P-related_33_10. One TerL sequence had 90% similarity to that of uv-Fonsiphage-EPL. *Fonsibacter* was detected in all 90 samples and showed an average rpS3 similarity of 99.6% to those from EPL/I-EPL/BMMRE samples (Fig. 4b). The *Fonsibacter* members accounted for 2.1 to 24.8% (10.8% on average; see Table S7 at https://figshare.com/articles/Supplementary_data_for_Chen_et_al_2019/9911318) of the bacterial communities (Fig. 4c), indicating that they were an important fraction of the indigenous microbiome. However, only 16 samples had a total phage relative abundance of ≥1% (two from 2010, the others from 2014) and up to 14.26% in the sample from 22 October 2012 (Fig. 4c; see Table S7 at https://figshare.com/articles/Supplementary_data_for_Chen_et_al_2019/9911318). There was no discernible pattern to explain the high Fonsiphage abundance in some samples. It should be noted that the calculated abundances were relative, not absolute, and thus could be influenced by the dynamics of the rest of the members in the communities.

In spite of their high TerL similarity, Lake Mendota phage showed different genomic features from EPL/I-EPL/BMMRE HTVC010P-related phage (see Fig. S7a at https://figshare.com/articles/Supplementary_data_for_Chen_et_al_2019/9911318). We reconstructed a complete 35,984-bp phage genome from the Lake Mendota data sets that includes 60 protein-encoding genes (Fig. 4d; also see Fig. S7b at https://figshare.com/articles/Supplementary_data_for_Chen_et_al_2019/9911318). Its TerL and major capsid proteins share 98.9% and 99.7% similarity to those of HTVC010P-related_32_16. This genome of the Lake Mendota phage has several genes not found in the EPL genome (Fig. 4d). It contained an HNH endonuclease that is present in HTVC010P-related_32_16 but absent in most HTVC010P-related phage (Fig. 2b). Phylogenetic analyses showed that these HTVC010P-related HNH endonucleases were closely related to those from uv-Fonsiphage-EPL and related phage (see Fig. S8 at https://figshare.com/articles/Supplementary_data_for_Chen_et_al_2019/9911318). However, we cannot distinguish whether the endonuclease was ancestral and lost in some members from acquisition via horizontal transfer.

### Wide distribution of Fonsiphage or Fonsiphage-like phage.

**FIG 5:**
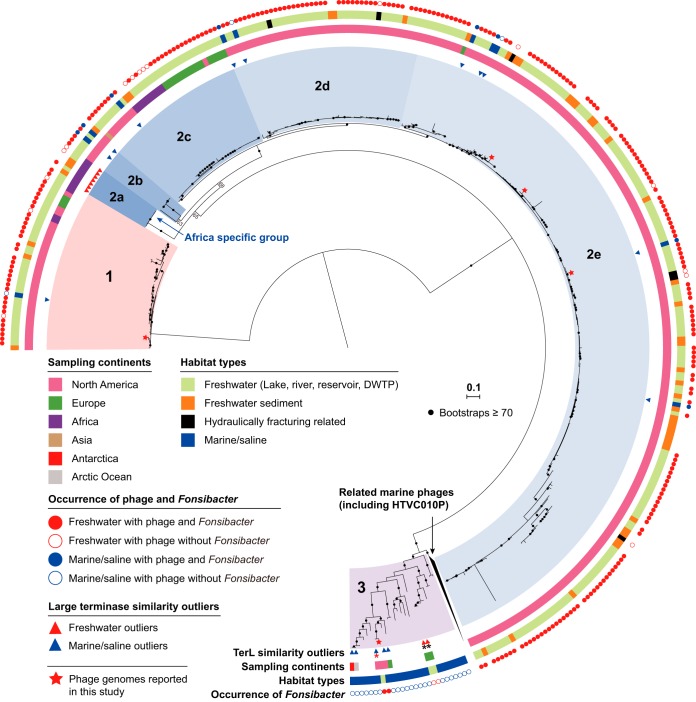
Phylogenetic analyses of HTVC010P-related phage in global freshwater ecosystems based on TerL. For phage from marine/saline habitats, only those with anomalously high similarity to the EPL/I-EPL/BMMRE phage were included, along with similar phage from marine group 3 as references. The similarity outliers from freshwater and related habitats and marine/saline habitats are indicated by red and blue triangles, respectively. The TerLs were assigned to habitat types and sampling continents based on sampling information. Stars represent phage whose genomes are reported in the current study. The phage were grouped into 3 groups based on the phylogeny and TerL identity (≥80%); subgroups were determined in group 2 based on phylogeny. The presence or absence of *Fonsibacter* in the sample with TerL detected is indicated by solid or open circles, respectively, and is not shown for sediment samples because none with *Fonsibacter* were detected. The TerLs detected in European eel-related samples are indicated by black asterisks, and the one from Lake Walker sediment is indicated by a red asterisk (in group 3). Bootstrap values are indicated by black dots if values are ≥70. DWTP, drinking water treatment plant.

By searching public metagenomic data sets (see Materials and Methods), we retrieved 403 TerL sequences from 193 freshwater-related samples and 2393 TerL sequences from 568 marine/saline samples (all shared ≥80% similarity to those of phage reported in this study [see Fig. S9a at https://figshare.com/articles/Supplementary_data_for_Chen_et_al_2019/9911318]). Overall, the freshwater-related TerL sequences shared on average 96% amino acid identity with those reported here, whereas marine/saline predicted proteins shared on average only 83% identity. However, some anomalously similar TerL sequences were detected in both habitat types (see Table S8 at https://figshare.com/articles/Supplementary_data_for_Chen_et_al_2019/9911318). Eleven of the freshwater-related outliers were from Africa inland freshwater lakes, including Kabuno Bay, Lake Kivu, and Lake Malawi, which are all geographically connected by the Rusizi River. Some of these phage cluster together in an Africa-specific group (group 2a [Fig. 5]) and apparently associate with an Africa-specific group of *Fonsibacter* (see Fig. S11 at https://figshare.com/articles/Supplementary_data_for_Chen_et_al_2019/9911318). The other two freshwater-related outliers were from the Alfacada pond (Ebro Delta, Spain) and associated with the skin of a European eel (23–25). For the marine/saline outliers, the majority were from San Francisco Bay, the Columbia River estuary, and the Delaware River and Delaware Bay, which are characterized by salinity gradients and thus could provide niches for *Fonsibacter* (see below).

We investigated the distribution of Fonsiphage or Fonsiphage-like phage in freshwater-related ecosystems using detection of the TerL protein sequence. The phage were detected in 118 lake/pond/reservoir, 35 river, 34 sediment, 4 hydraulic-fracturing-related, and two drinking water treatment plant samples (see Fig. S9b and Table S8 at https://figshare.com/articles/Supplementary_data_for_Chen_et_al_2019/9911318). *Fonsibacter* was found in most samples but not in the freshwater sediment and three hydraulic-fracturing-related samples (see Fig. S9b at https://figshare.com/articles/Supplementary_data_for_Chen_et_al_2019/9911318). Phage related to HTVC010P-related_32_16 (groups 2a to e [Fig. 5]) and HTVC010P-related_33_10 (group 1 [Fig. 5]) were widely distributed. However, HTVC010P-related_33_76 (group 3 [Fig. 5]) and uv-Fonsiphage-EPL phage were detected in only two and six freshwater habitats (see Fig. S10 at https://figshare.com/articles/Supplementary_data_for_Chen_et_al_2019/9911318), respectively. With the exception of our study and the two European eel-associated samples, the HTVC010P-related_33_76 phage group was never detected in other freshwater-related habitats (Fig. 5; see also Table S8 at https://figshare.com/articles/Supplementary_data_for_Chen_et_al_2019/9911318).

## DISCUSSION

### *Fonsibacter* phage are widely distributed but show regional diversification.

We reconstructed genomes of one temperate and five lytic Fonsibacter phage (Table 1 and Fig. 4d) that are very similar to those of some Pelagiphage (Fig. 1, 2, and 4d). Among them, uv-Fonsiphage-EPL is the only reported prophage genome of *Fonsibacter* so far; the detection of lysis-related genes indicates that it could affect the infected *Fonsibacter* population. Given that uv-Fonsiphage-EPL lacks certain hypothetical proteins found in most *HTVC019Pvirus* group III phage, we conclude that these proteins are not necessary for infection or replication in *Fonsibacter*.

Most of the lytic HTVC010P-related phage were widely distributed but show evidence of regional diversification (Fig. 5). For example, groups 1 and 2c were detected in at least 3 continents. Phage from the same continent tend to be more closely phylogenetically related. This suggests the existence of barriers that inhibit dispersal of most groups, possibly related to variations in indigenous phage resistance. Alternatively, the current distribution patterns of groups 1 and 2c may reflect several recent, independent transitions of phage and their hosts from marine to terrestrial freshwater environments, without time for wider dispersal across continents. However, another factor could be sampling bias, as most of the samples analyzed were from North America (Fig. 5).

The cooccurrence of HTVC010P-related phage and *Fonsibacter* suggests a stable host-phage relationship. However, despite the presence of *Fonsibacter* phage, we did not identify *Fonsibacter* in freshwater sediment or three out of the four hydraulic-fracturing-related samples (Fig. 5; see also Fig. S11 at https://figshare.com/articles/Supplementary_data_for_Chen_et_al_2019/9911318) or in one sample from the EPL water-sediment interface sample. The phage in the sediment samples could have settled from the overlying water column. A similar process may explain the presence of phage that shared identical TerL sequences in both the freshwater and sediment Lake Kivu samples (see Table S8 at https://figshare.com/articles/Supplementary_data_for_Chen_et_al_2019/9911318).

### Multiple marine-freshwater transitions of *Fonsibacter* phage.

The similarity between the *Fonsibacter* phage and Pelagiphage (Fig. 1 and 2) indicates that they share an ancestor. A question is whether *Fonsibacter* phage transitioned from marine environments only once or multiple times. We detected groups 1 and 2a to -e of the HTVC010P-related phage in many freshwater habitats and some environments with freshwater-to-saline gradients (Fig. 5; see also Table S8 at https://figshare.com/articles/Supplementary_data_for_Chen_et_al_2019/9911318). However, the majority of group 3 of HTVC010P-related phage were from marine habitats, with only three TerL sequences from two freshwater habitats (Fig. 5). On the other hand, detection of HTVC010P-related_33_76 at high relative abundance in EPL during an 8-month sampling period (Fig. 3b; see also Fig. S6 at https://figshare.com/articles/Supplementary_data_for_Chen_et_al_2019/9911318) indicates persistence in this habitat. Moreover, these four freshwater phage clustered into two distinct groups (Fig. 5), possibly indicating that they originated from different marine phage genotypes. Also, the high genomic similarity of uv-Fonsiphage-EPL to its marine relatives (10, 12) indicated another transition of *Fonsibacter* phage from marine to freshwater habitats. However, the limited distribution of uv-Fonsiphage-EPL-related phage (see Fig. S10 at https://figshare.com/articles/Supplementary_data_for_Chen_et_al_2019/9911318) likely suggested the existence of an unknown biotic/abiotic barrier to their spread.

### Conclusions.

The persistent inertia in culturing freshwater microbes challenges our understanding of the ecology and functions of aquatic ecosystems. Genome-resolved metagenomics is a promising approach to solve this problem, by reconstructing complete genomes of bacterial hosts and their infecting phage. In this study, we report complete genomes of *Fonsibacter* and both lysogenic and lytic infecting phage, revealing their similarity to marine Pelagiphage and their wide distribution in freshwater habitats. Based on this, more detailed analysis on the interaction of *Fonsibacter* and infecting phage could be performed in future studies.

## MATERIALS AND METHODS

### Sampling, DNA extraction, sequencing, metagenomic assembly, and genome binning.

The EPL samples were collected from an end pit lake (EPL) in Alberta, Canada, in 2017 (15 samples) and 2018 (2 samples), at multiple depths (see Table S1 at https://figshare.com/articles/Supplementary_data_for_Chen_et_al_2019/9911318). Also, one sample was collected from the input source of EPL (I-EPL) on 19 September 2017 at a depth of 0.5 m (I-EPL_09192017_0.5m). The BMMRE samples were collected from a base metal mine receiving environment in northern Manitoba, Canada, in 2016 (July 24) and 2017 (April 13 and September 27). The geochemical features of the samples were determined *in situ* or in the laboratory as previously described (26).

Genomic DNA was collected by filtering ca. 1.5 liters of water through 0.22-μm Rapid-Flow sterile disposable filters (Thermo Fisher Scientific) and stored at −20°C until the DNA extraction. DNA was extracted from the filters as previously described (27). The DNA samples were purified for library construction and sequenced on an Illumina HiSeq1500 platform with paired-end (PE) 150-bp kits. The raw reads of each metagenomic sample were filtered to remove Illumina adapters, PhiX, and other Illumina trace contaminants with BBTools (28), and low-quality bases and reads were removed using Sickle (version 1.33; https://github.com/najoshi/sickle). The high-quality reads of each sample were assembled using idba_ud (29) (parameters: –mink 20 –maxk 140 –step 20 –pre_correction). For a given sample, the high-quality reads of all samples from the same sampling site were individually mapped to the assembled scaffold set of each sample using Bowtie 2 with default parameters (30). The coverage of the scaffold was calculated as the total number of bases mapped to it divided by its length. Multiple coverage values were obtained for each scaffold to reflect the representation of that scaffold in the various samples. For each sample, scaffolds with a minimum length of 1.5 kbp were assigned to preliminary draft genome bins using MetaBAT with default parameters (31), with both tetranucleotide frequencies (TNF) and coverage profile of scaffolds considered. The scaffolds from the obtained bins and the unbinned scaffolds with a minimum length of 1 kbp were uploaded to ggKbase (https://ggkbase.berkeley.edu/). The genome bins detected with *Fonsibacter-*related scaffolds were evaluated based on the consistency of GC content, coverage, and taxonomic information, and scaffolds identified as contaminants were removed.

### Manual curation of *Fonsibacter* and phage genomes.

The *Fonsibacter* genome bin with 27 scaffolds was manually curated to completion, by performing first an overlap-based assembly of scaffolds using Geneious (32) and then linkage of scaffolds by metaSPAdes-assembled scaffolds and scaffold extension and manual fixation of local assembly errors detected by ra2.py (33). A total of 51 bacterial universal single-copy genes (SCGs) were used to evaluate genome completeness (34). One prophage was detected in the complete *Fonsibacter* genome. This prophage was manually curated into a circular genome using paired-end reads located at both ends of the prophage region in the host genome. To obtain genomes of potential *Fonsibacter*-infecting phage, we identified the EPL/I-EPL/BMMRE scaffolds with multiple genes closest to those of published Pelagiphage. For these scaffolds, manual curation including assembly error fixation was performed (using the same methods as for the *Fonsibacter* genome). To investigate (and for reference) how the circular genome of the phage relates to the prophage sequence, see the step-by-step procedures at https://figshare.com/articles/Supplementary_data_for_Chen_et_al_2019/9911318.

The protein-encoding genes of the curated *Fonsibacter* and phage genomes were predicted using Prodigal (35) and searched against KEGG, UniRef100, and UniProt for annotation, and metabolic pathways were reconstructed. The 16S rRNA gene of *Fonsibacter* was predicted based on the HMM model as previously described (33). The tRNAs in *Fonsibacter* and phage genomes were predicted using tRNAscan-SE 2.0 (36). The transmembrane domains and signal peptide of proteins were predicted using Phobius (37). The identification of CRISPR-Cas systems in assembled scaffolds was performed using a Python script (https://github.com/linxingchen/CRISPR); all unique CRISPR spacers were extracted from the scaffolds, and reads were mapped to the scaffolds and searched against the curated phage genomes for the potential target using BLASTn (BLASTn-short). ANI was calculated using the online tool OrthoANIu (38).

For comparative genomic analyses of phage related to uv-Fonsiphage-EPL, we included all the published *HTVC019Pvirus* Pelagiphage as analyzed in reference 12. For comparative analyses of HTVC010P-related phage, we searched the TerL proteins against NCBI-nr using BLASTp, and NCBI scaffolds/genomes having a hit with ≥70% similarity (few with ≥80% similarity) were retained for further analyses (see Table S2 at https://figshare.com/articles/Supplementary_data_for_Chen_et_al_2019/9911318). The predicted proteins of the selected NCBI scaffolds/genomes were downloaded from NCBI, and protein family analyses were performed as previously described (39), including the proteins of newly constructed phage genomes. In detail, first, all-versus-all searches were performed using MMseqs2 (40), with parameters set as E value = 0.001, sensitivity = 7.5, and cover = 0.5. Second, a sequence similarity network was built based on the pairwise similarities, and then the greedy set cover algorithm from MMseqs2 was performed to define protein subclusters (i.e., protein subfamilies). Third, in order to test for distant homology, we grouped subfamilies into protein families using an HMM-HMM comparison procedure as follows. The proteins of each subfamily with at least two protein members were aligned using the result2msa parameter of MMseqs2, and HMM profiles were built from the multiple sequence alignment using the HHpred suite (41). The subfamilies were then compared to each other using hhblits (42) from the HHpred suite (with parameters -v 0 -p 50 -z 4 -Z 32000 -B 0 -b 0). For subfamilies with probability scores of ≥95% and coverage of ≥0.5, a similarity score (probability × coverage) was used as the weights of the input network in the final clustering using the Markov CLustering algorithm (43), with 2.0 as the inflation parameter. Finally, the resulting clusters were defined as protein families.

### Phylogenetic analyses.

Multiple phylogenetic trees based on different genes (or gene sets) were built in this study (some are described at https://figshare.com/articles/Supplementary_data_for_Chen_et_al_2019/9911318).

### (i) Sixteen rp’s of SAR11 genomes.

For reference, SAR11 genomes at NCBI were downloaded and evaluated using CheckM to filter those genomes with completeness lower than 70%. The 16 ribosomal proteins (rp’s) (i.e., L2, L3, L4, L5, L6, L14, L15, L16, L18, L22, L24, S3, S8, S10, S17, and S19) were predicted from the NCBI genomes and the *Fonsibacter* genomes from this study, using HMM-based search as previously described (34). Those genomes with none (AAA024-N17, AAA023-L09, AAA027-L15) or only two (AAA280-B11) of these 16 rp’s were excluded for analyses.

### (ii) rpS3.

The ribosomal protein S3 (rpS3) marker gene was used to identify *Fonsibacter* in metagenomic data sets and also for phylogenetic analyses using the nucleotide sequences.

### (iii) Concatenated proteins of phage.

Via protein family analyses (see above), the 12 core proteins detected in the 28 uv-Fonsiphage-EPL-related phage were used for phylogenetic analyses (see Table S3 at https://figshare.com/articles/Supplementary_data_for_Chen_et_al_2019/9911318; two genomes lack one of the 12 core proteins due to incompleteness) (12).

### (iv) TerL.

The phage large terminase was used for several phylogenetic analyses, including the HTVC010P-related phage analyses and those for the phage detected in Lake Mendota (clustered with 99% identity) and the phage identified in other habitats worldwide.

For tree construction, protein sequence data sets were aligned using Muscle (44). All the alignments were filtered using trimAL (45) to remove those columns comprising more than 95% gaps and also ambiguously aligned C and N termini. For the 16 ribosomal proteins and the 12-phage-protein sets, sequences were concatenated into a single aligned sequence. The phylogenetic trees (including concatenated and TerL) were constructed using RAxML version 8.0.26 with the following options: -m PROTGAMMALG -c 4 -e 0.001 -# 100 -f a (46). For rpS3, the nucleotide sequences were aligned and filtered as described above, and the tree was built using RAxML version 8.0.26 with the following options: -m GTRGAMMAI -c 4 -e 0.001 -# 100 -f a (46). All the trees were uploaded to iTOL v3 for visualization and formatting (47).

### Codon usage analyses and cooccurrence of *Fonsibacter* and phage.

The codon usage frequency of phage, bacterial, and archaeal genomes was determined using the Cusp (create a codon usage table) program of EMBOSS (The European Molecular Biology Open Software Suite), with protein-encoding genes predicted by Prodigal (-m single, translation table 11). The prophage region in *Fonsibacter*_30_26 was removed from the host genome before performing gene prediction. Clustering analyses of all these genomes based on their codon usage frequency were performed using the R package of Pheatmap (48), with Euclidean clustering and average method (Fig. 3a). The usage frequency of each synonymous codon for a given amino acid is listed in Table S5 at https://figshare.com/articles/Supplementary_data_for_Chen_et_al_2019/9911318. To evaluate the occurrence of *Fonsibacter* and phage in the EPL/I-EPL/BMMRE samples (Fig. 3b), we used the rpS3 gene to identify *Fonsibacter* (and also the three genomes with similar codon usage frequencies) and the TerL gene to identify phage. The genotype was determined based on sharing ≥99% phage TerL or *Fonsibacter* rpS3 amino acid similarity.

### Analyses of published data from Lake Mendota.

*Fonsibacter* was studied previously in Lake Mendota (3, 22). The published metagenomic data sets deposited at Integrated Microbial Genomes (IMG) were searched for phage similar to the ones from EPL/I-EPL/BMMRE samples, using their TerL proteins as queries. We also obtained the rpS3 protein sequences from these data sets using BLASTp at IMG and HMM-based confirmation using the TIGRFAM database (49). The rpS3 proteins belonging to *Fonsibacter* were identified by phylogenetic analyses with all available SAR11 rpS3 sequences.

Raw paired-end reads of the time-series Lake Mendota samples were downloaded from the NCBI SRA via the information provided in reference 22. A total of 90 data sets were available for download. Quality control was performed on those raw reads as described above. To determine the relative abundance of both *Fonsibacter* and phage in each sample, we first mapped quality reads of each sample to all confirmed and nonredundant (clustered at 100% identity) rpS3 genes from the 90 Lake Mendota samples and then filtered the mapping file to allow no more than 3 mismatches for each read (equal to 98% similarity). The coverage of each rpS3 gene across all 90 samples was determined as described above. For a given sample, the total relative abundance of *Fonsibacter* was determined by first summing the total coverage of all *Fonsibacter* rpS3 genes (referred to as *a*) and the total coverage of all bacterial and archaeal rpS3 genes (referred to as *b*), and then the accumulated relative abundance of *Fonsibacter* in this sample was calculated as *a*/*b* × 100%. To have a similar evaluation of the relative abundance of *Fonsibacter* phage in the 90 Lake Mendota samples, we did the mapping, filtering, and coverage calculation for the detected nonredundant (clustered at 100% identity) TerL genes related to the *Fonsibacter* phage from EPL/I-EPL/BMMRE. In a given sample, the total relative abundance of *Fonsibacter* phage was determined by first summing their accumulated coverage based on the TerL genes (referred to as *c*) and then calculated as *c*/*b* × 100%.

We performed *de novo* assembly using idba_ud on the data sets from October 2012, in which the HTVC010P-related phage had a high coverage. BLAST (including BLASTp and BLASTn) was used to retrieve scaffolds similar to HTVC010P-related phage from the assembled data sets, followed by manual curation and resulting in one complete phage genome. Gene prediction and annotation were conducted as described above.

### Global search of similar phage in IMG metagenomic data sets.

With the TerL proteins of uv-Fonsiphage-EPL and HTVC010P-related phage reported in this study as queries, a global search was performed against the metagenomic data sets in the IMG system using BLASTp. The hits were filtered with a minimum BLAST alignment coverage of 80% and a minimum similarity of 80%. We also searched for *Fonsibacter* in the metagenomic data sets with phage TerL detected, using the rpS3 protein sequences from all available *Fonsibacter* genomes as queries. The resulting hits were filtered for ≥80% alignment coverage and ≥80% similarity (it was preliminarily determined that a given rpS3 with similarity of <85% to *Fonsibacter* rpS3 is not a *Fonsibacter* rpS3), and then a phylogenetic tree was built to retrieve the sequences assigned to the *Fonsibacter* subclade. To report the BLASTp hits in the IMG metagenomic data sets in this work, we asked the principal investigators for each public data set for their permission to report the results (see Table S8 at https://figshare.com/articles/Supplementary_data_for_Chen_et_al_2019/9911318). The data in these metagenomic data sets have been published (50–56) or are in preparation for publication.

To compare the relationships of the related phages identified in this study and from IMG freshwater habitats, phylogenetic analyses based on TerL were performed. We also included the TerLs detected as similarity outliers and those related to HTVC010P-related_33_76 from IMG marine/saline habitats. The TerL sequences were dereplicated from each sampling site using cd-hit (-c 1 -aS 1 -aL 1 -G 1). Then, the representatives and the TerL from HTVC010P-related phage genomes reported in this study, and also those of HTVC010P and related marine phage (as references), were aligned and filtered for tree building (see “Phylogenetic analyses”). Another tree was built for the TerL of uv-Fonsiphage-EPL and relatives, with the same procedure as described above.

To evaluate the phylogeny and diversity of *Fonsibacter* in the samples with TerL detected and analyzed, phylogenetic analyses based on the rpS3 nucleotide sequences of *Fonsibacter* were performed. We also included all the *Fonsibacter* rpS3s identified in EPL/I-EPL/BMMRE samples, with rpS3 from SAR11 marine and brackish subclades as references. All the sequences were aligned and filtered for tree building (see “Phylogenetic analyses” above).

### Data availability.

The *Fonsibacter* and phage genomes have been deposited at NCBI under BioProject accession no. PRJNA552483. Besides being publicly accessible data at NCBI, the genomes are also available at https://figshare.com/articles/Fonsibacter_and_phages_genomes/9867587. All supplementary tables, figures, and information are available at https://figshare.com/articles/Supplementary_data_for_Chen_et_al_2019/9911318.
